# Prognostic Analysis of Lung Adenocarcinoma Based on DNA Methylation Regulatory Factor Clustering

**DOI:** 10.1155/2021/1557968

**Published:** 2021-08-26

**Authors:** Yang Chen, Caiming Zhong, Shujun Bao, Zheng Fang, Hao Tang

**Affiliations:** Department of Respiratory and Critical Care Medicine, Changzheng Hospital, Naval Medical University, Shanghai, China

## Abstract

There is a known link between DNA methylation and cancer immunity/immunotherapy; however, the effect of DNA methylation on immunotherapy in lung adenocarcinoma (LUAD) remains to be elucidated. In the current study, we aimed to screen key markers for prognostic analysis of LUAD based on DNA methylation regulatory factor clustering. We classified LUAD using the NMF clustering method, and as a result, we obtained 20 DNA methylation regulatory genes. These 20 regulatory genes were used to determine the pattern of DNA methylation regulation, and patients were grouped for further analysis. The risk score model was analyzed in the TCGA dataset and an external validation set, and the correlation between the risk score and DNA methylation regulatory gene expression was explored. We analyzed the correlation between the prognostic model and immune infiltration and checkpoints. Finally, we analyzed the Gene Ontology and Kyoto Encyclopedia of Genes and Genomes functions of the prognosis model and established the nomogram model and decision tree model. The survival analyses of ClusterA and ClusterB were significantly different. Survival analysis showed that patients with a high risk score had a poor prognosis. Survival models (tobacco, T, N, M, stage, sex, age, status, and risk score) were abnormally correlated with T cells and macrophages. The higher the risk score associated with smoking was and the higher the stage was, the more severe the LUAD and the more maladjusted the immune system were. Immune infiltration and abnormal expression of immune checkpoint genes in the prognostic model of LUAD were associated with the risk score. The prognostic models were mainly enriched in the cell cycle and DNA replication. Characterization of DNA methylation regulatory patterns is helpful to improve our understanding of the immune microenvironment in LUAD and to guide the development of a more personalized immunotherapy strategy in the future.

## 1. Introduction

Lung adenocarcinoma (LUAD) is the most common histological subtype of primary lung cancer. LUAD is usually evolved from mucosal glands, accounts for approximately 40% of all lung cancer cases, and is one of the most aggressive and rapidly lethal tumor types [1]. LUAD is usually diagnosed as disseminated metastatic tumors at an advanced stage, with an overall survival rate of <5 years [2]. As a heterogeneous disease, LUAD has important therapeutic significance [3]. In many cases, LUAD can be found in areas of scarring or chronic inflammation [4]. Studies have shown that FDG PET can be used to predict the histopathological classification and growth pattern of early LUAD [5]. Although LUAD can be surgically resected, approximately half of patients with early lung cancer who undergo resection eventually relapse and die of the disease [6]. Epigenetic changes are considered potential biomarkers for the early diagnosis of various cancer tissue types, and novel DNA methylation markers have been reported to play an important role in the early diagnosis of LUAD [7]. Therefore, it is important to explore the role of DNA methylation in the pathogenesis of LUAD.

DNA methylation is abnormal in cancer and is often described as a “silent” epigenetic marker [8, 9]. Changes in DNA methylation may lead to changes in gene expression profiles, most notably promoter DNA hypermethylation leading to silencing of tumor suppressors, microRNAs, endogenous retroviruses, and tumor antigens, as well as genomic DNA hypermethylation leading to the upregulation of oncogenes [10]. The potential of epigenetic-targeted drugs in combination with chemotherapy, targeted therapy, and/or immunotherapy is being investigated [11]. An association between DNA methylation and tumor immunity/immunotherapy has been reported [12], and changes in DNA methylation are associated with epigenetic regulation in precision immunotherapy [13]. However, the role of DNA methylation in LUAD immunopathology and immunotherapy remains unclear.

The immune system plays an active role in the occurrence and growth of LUAD [14]. Tumor-infiltrating immune cells have been shown to be positively correlated with overall survival (OS) in patients with LUAD [15]. The tumor immune microenvironment (TIM) plays a key role in the regulation of tumor progression, and the rapid development of immunotherapy has opened up a new approach for cancer treatment. Immune checkpoint blockade therapy has revolutionized the treatment of human cancers, including LUAD [16]. It has been reported that a large number of patients with advanced LUAD have targeted mutations [17]. In these patients, antibodies against immune checkpoints, such as programmed death-1 (PD-1) and cytotoxic T-lymphocyte-associated antigen-4 (CTLA-4), have demonstrated the safety of treatment [18]. This reflects the importance of the TIM to the clinical outcomes of patients with LUAD. Therefore, greater understanding of the immune microenvironment of LUAD will serve to guide immunotherapy strategies.

In this study, we classified LUAD using the NMF clustering method and, as a result, obtained 20 DNA methylation regulatory genes to determine the pattern of DNA methylation regulation. We investigated the association between the risk score and DNA methylation regulatory gene expression and analyzed the correlation between the prognostic model and immune infiltration and checkpoints. Finally, we analyzed the Gene Ontology (GO) and Kyoto Encyclopedia of Genes and Genomes (KEGG) functions of the prognosis model and established the nomogram model and decision tree model. Our results showed that the more the risk score was associated with smoking and the higher the stage was, the more severe the disease was and the more the immune system was maladjusted in patients with LUAD. Immune infiltration and abnormal expression of immune checkpoint genes in the prognostic model of LUAD were associated with the risk score. The characterization of the regulatory pattern of DNA methylation is helpful to guide the development of a more personalized immunotherapy strategy in the future.

## 2. Materials and Methods

### 2.1. LUAD Datasets and Preprocessing

The TCGA dataset was downloaded from UCSC Xena (https://xenabrowser.net/), and RNA sequencing (RNA-Seq) data were downloaded from the TCGA data portal. The fragment's value per million fragment numbers (FPKM) was then converted into the transcript per million byte points (TPM) value. The microarray dataset GSE72094 served as an external validation set from the Gene Expression Omnibus (https://www.ncbi.nlm.nih.gov/geo/). Raw data from the microarray dataset were generated by Affymetrix. The RMA algorithm in the Affy software package was then applied to process the raw data from Affymetrix for quantile normalization and background correction. All data were analyzed using R software (version 3.6.1) and the R Bioconductor software package. A total of 500 samples were included, and clinicopathological data were collected, including tobacco, T, N, M, stage, gender, age, and status (Table 1).

### 2.2. Clustering Types of DNA Methylation Regulatory Genes

LUAD was classified using the NMF clustering method, and 20 DNA methylation regulatory genes were obtained to determine the pattern of DNA methylation regulation. The patients were then grouped for further analysis.

### 2.3. Establishment of the Risk Score for DNA Methylation Regulation

The “limma” package in R software was used to identify differentially expressed genes (DEGs) associated with two immune checkpoint-related patterns [19]. *P* values <0.05 and |logFC| > log2 (1.5) were used to determine significant sexual criteria of DEGs. Univariate Cox regression analysis was used to determine the representative DEG, and then lambda values (lambda-1se) were selected through 1,000 cross-validations using the lasso method. A set of prognostic genes and their lasso regression coefficients were obtained. The risk score was the sum of the expression value of the genes screened by lasso, and the risk score was divided into two groups of high and low risk by the optimal point method. Risk score = 0.1798 *∗* UHRF1 + −0.1435 *∗* CLSPN + 0.2179 *∗* CENPE + −0.2046 *∗* POLQ + −0.06 *∗* MCM4 + 0.0325 *∗* BRIP1 + −0.1421 *∗* HELLS + −0.0996 *∗* ATAD2 + 0.138 *∗* ZNF367 + −0.2166 *∗* ESCO2 + 0.1384 *∗* TMPO + 0.2106 *∗* POP1 + 0.1902 *∗* NUP107 + 0.1177 *∗* FXYD1 + −0.0408 *∗* GGTLC1 + 0.0288 *∗* HIF1A + −0.0186 *∗* LDHD + −0.2112 *∗* MUSTN1 + −0.0533 *∗* GPD1L + 0.0408 *∗* TDRD10 + −0.0311 *∗* TMEM130 + −0.0908 *∗* FBP1 + −0.0839 *∗* ATAD3C + 0.1504 *∗* IL1R2.

### 2.4. Estimation of Immune Infiltration

The CIBERSORT, ESTIMATE, MC, and TIMER algorithms were compared to assess the relationship between the risk score and cell composition or cellular immune response, and the differences in the immune response under different algorithms were revealed using heat maps [20].

### 2.5. Pathway Analysis

We calculated the risk score and all of the correlations of the gene expression of the relativity of selected gene enrichment analysis, mainly analyzed the GO of biological processes (BP), cellular components (CC), molecular function (MF), and KEGG. The definition of correlation was |cor| >0.3 and *P* < 0.05.

### 2.6. Establishment of the Nomogram

According to the results of the multivariate Cox regression analysis, a nomogram was constructed using “RMS” in R software to predict 1-, 3-, and 5-year OS. The prediction accuracy of the OS nomogram model was evaluated by the calibration curve and DCA.

### 2.7. Statistical Analysis

The Shapiro–Wilk normality test was used to check the normality of variables. For normally distributed variables, an unpaired Student's *t*-test was used to compare the differences between the two groups. The Wilcoxon test was used to compare variables that were not normally distributed. Pearson correlation and distance correlation analyses were used to calculate the correlation coefficients. Patients were divided into high or low risk scores of each dataset according to the risk score of dichotomy. The data were mainly visualized using the R package “ggplot2.” The Benjamini–Hochberg method was used to analyze DEGs, which converted *P* values into FDR to identify important genes. The Kaplan–Meier method was used to generate and visualize subgroup survival curves, and the log-rank test was used to determine the statistical significance of differences in each dataset. The “rpart” package was used for decision tree analysis. All survival curves were generated by the R package “survminer.” All heat maps were generated based on “pheatmap.” All statistical analyses were performed in R (https://www.r-project.org/, version 3.6.1). All of the tests were two-sided, and *P* values <0.05 were considered statistically significant.

## 3. Results

### 3.1. DNA Methylation Regulates Gene Clustering

**Figure 1:**
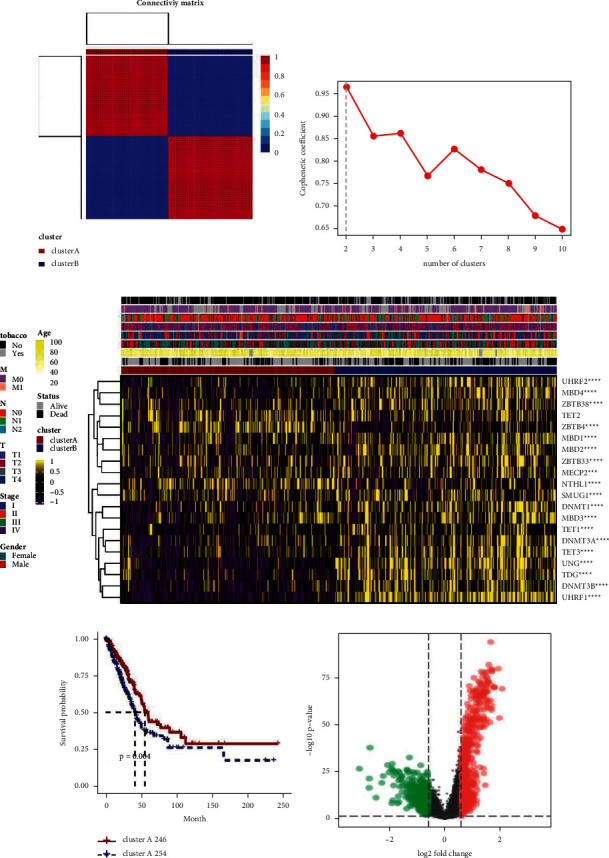
DNA methylation regulates gene clustering. (a, b) The optimal clusters were ClusterA and ClusterB according to DNA methylation regulation clustering. (c) Total DNA methylation regulatory genes and their clinical characteristics in ClusterA and ClusterB. (d) Survival analyses of ClusterA and ClusterB. (e) Volcano maps of ClusterA and ClusterB differential genes. ^*∗∗∗∗*^*P* < 0.0001.

First, we constructed the connectivity matrix, which was based on DNA methylation regulatory clustering and was optimized to cluster into two classes: ClusterA and ClusterB. The cophenetic coefficient decreased with the increase in the number of clusters (Figures 1(a) and 1(b)). Subsequently, LUAD was classified using the NMF clustering method, and 20 DNA methylation regulatory genes were obtained to determine the pattern of DNA methylation regulation. All DNA methylation regulatory genes and their clinical characteristics in ClusterA and ClusterB are shown in Figure 1(c). UHRF2, MBD4, ZBTB38, ZBTB4, MBD1, MBD2, ZBTB33, MECP2, NTHL1, SMUG1, DNMT1, MBD3, TET1, DNMT3A, TET3, UNG, TDG, DNMT3B, and UHRF1 were associated with tobacco, T, N, M, stage, sex, age, and status. Survival analyses were conducted on ClusterA and ClusterB, and the results showed significant differences between the two groups (Figure 1(d)). Figure 1(e) shows the volcano map of the two types of differential genes. These results showed that there were significant differences between ClusterA and ClusterB.

### 3.2. Establishment and Verification of Prognosis Scores

**Figure 2:**
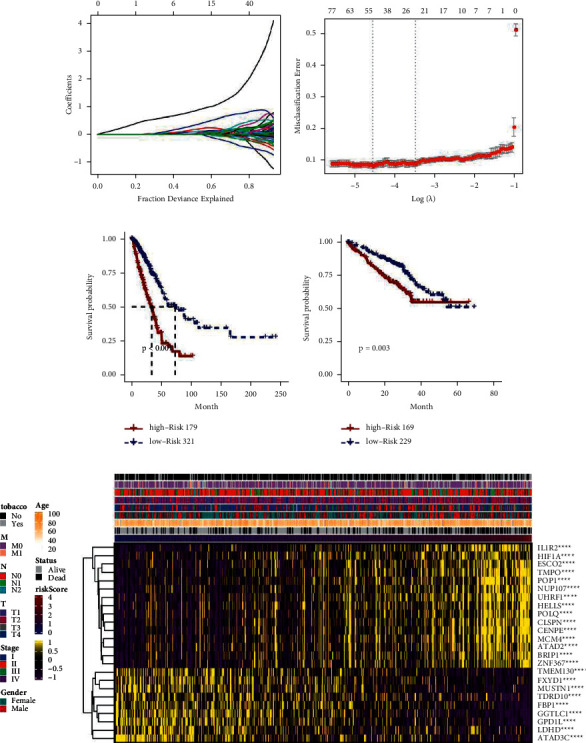
Establishment and verification of prognosis scores. (a, b) Risk score model of 24 genes. (c, d) Survival analysis of the TCGA dataset and the risk score model of the external validation set. (e) Demonstration of DNA methylation regulatory genes and clinical characteristics as the risk score increased from low to high. ^*∗∗∗∗*^*P* < 0.0001.

The limma package in R software was used to identify the DEGs associated with the two immune checkpoint-related patterns, and the number of DEGs in the two categories was 1026. A total of 607 genes were screened by univariate analysis in the TCGA dataset, and a risk score model containing 24 genes was screened by the lasso machine learning method (Figures 2(a) and 2(b)). The risk score model was analyzed in the TCGA dataset and the external validation set, as shown in Figures 2(c) and 2(d). Survival analysis showed that patients with high risk scores had poor prognosis (*P* < 0.05). We then explored the relationship between the risk score and DNA methylation regulation of gene expression.  Figure 2(e) shows that the survival model (tobacco, T, N, M, stage, sex, age, status, and risk score) was associated with IL1R2, HIF1A, ESCO2, TMPO, POP1, NUP107, UHRF1, HELLS, POLQ, CLSPN, CENPE, MCM4, ATAD2, BRIP1, ZNF367, TMEM130, FXYD1, MUSTN1, TDRD10, FBP1, GGTLC1, GPD1L, LDHD, and ATAD3C.

### 3.3. Immune Infiltration and Immune Checkpoint Analysis of the Prognostic Model

**Figure 3:**
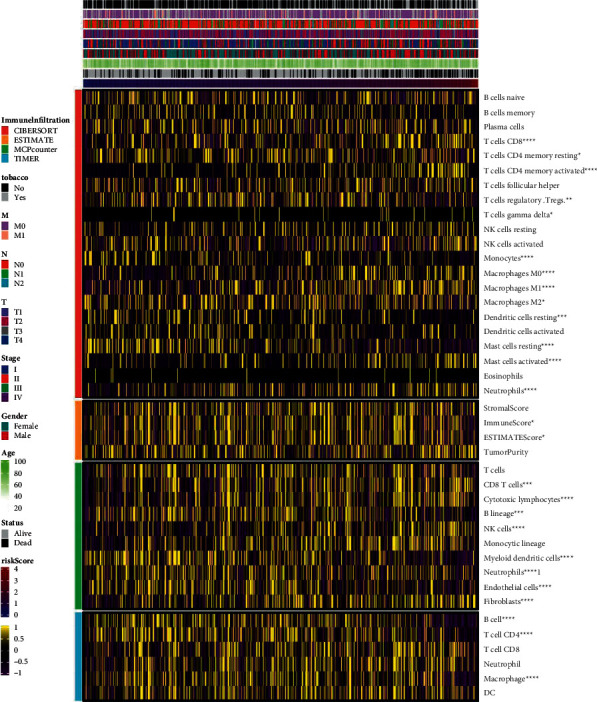
Correlation analysis of immune infiltration among different immune infiltration algorithms as risk scores ranged from low to high. ^*∗*^*P* < 0.05, ^*∗∗*^*P* < 0.01, ^*∗∗∗*^*P* < 0.001, and ^*∗∗∗∗*^*P* < 0.0001.

**Figure 4:**
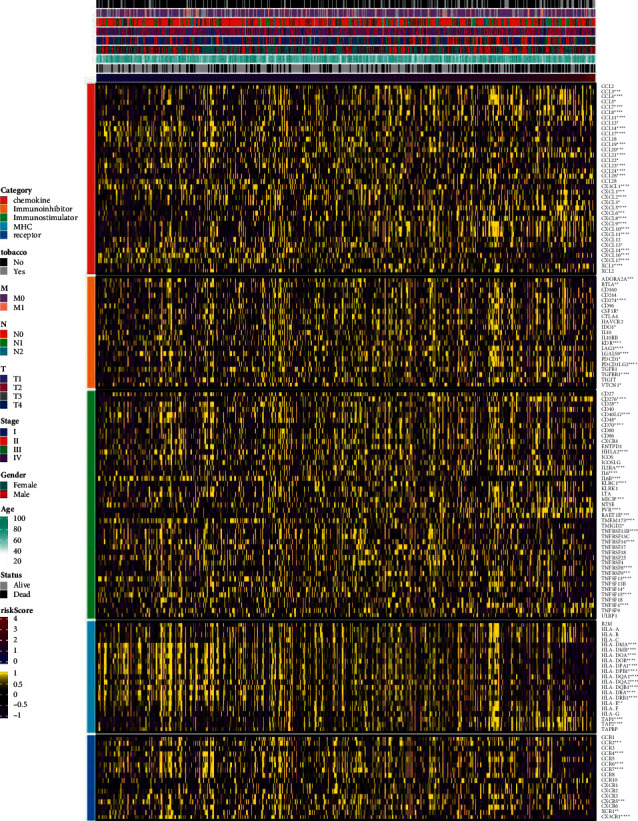
Correlation analysis of different immune regulatory factors from low to high risk scores. ^*∗*^*P* < 0.05, ^*∗∗*^*P* < 0.01, ^*∗∗∗*^*P* < 0.001, and ^*∗∗∗∗*^*P* < 0.0001.

Next, we analyzed the correlation between prognosis and immune infiltration.  Figure 3 shows the relationship between the risk score and cell composition or cellular immune response under CIBERSORT, ESTIMATE, McCounter, and TIMER algorithms. Heat maps revealed differences in the immune response under different algorithms. Under the CIBERSORT algorithm, CD8 T cells, CD4 memory-activated T cells, monocytes, M0 macrophages, M1 macrophages, resting mast cells, activated mast cells, and neutrophils were abnormal. Under the McCounter algorithm, cytotoxic lymphocytes, NK cells, myeloid dendritic cells, neutrophils, endothelial cells, and fibroblasts were abnormal. Under the TIMER algorithm, B cells, CD4 T cells, and macrophages were abnormal. In general, survival models (tobacco, T, N, M, stage, sex, age, status, and risk score) were abnormally correlated with T cells and macrophages. The higher the risk score associated with smoking and the higher the stage, the more severe the LUAD and the more maladjusted the immune system. These results suggested that immune infiltration was associated with a risk score in the prognostic model of LUAD. The correlation analysis of different immune regulatory factors when the risk score changed from low to high is shown in Figure 4. The immune checkpoint categories are as follows: chemokine, immune inhibitor, immune stimulator, and MHC and receptor. Among them, the higher the risk score associated with smoking and the higher the stage, the more severe the LUAD. In addition, the abnormal expression of immune checkpoint genes was significantly correlated with high and low risk scores.

### 3.4. Functional Analysis of the Prognostic Model

**Figure 5:**
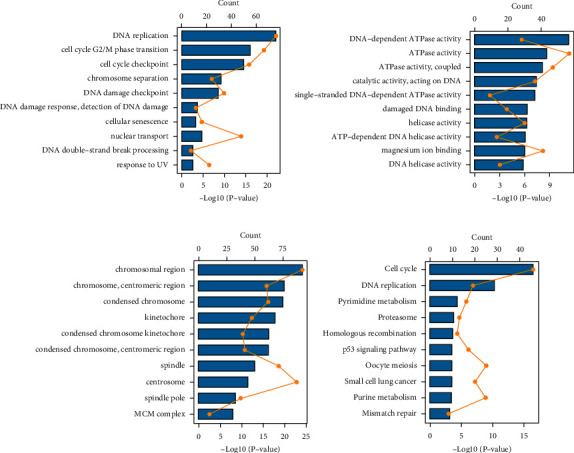
Functional analysis of the prognostic model. (a) Biological process in GO. (b) Cellular component in GO. (c) Molecular function in GO. (d) KEGG pathway.

We next performed functional analysis to further investigate the prognostic model. The BP in GO analysis is shown in Figure 5(a). It mainly involved DNA replication, cell cycle G2-/M-phase transition, and cell cycle checkpoint. The CC in GO analysis mainly involved DNA-dependent ATPase activity and ATPase activity (Figure 5(b)). MF in GO analysis mainly referred to the chromosonmal region, chromosome, centromeric region, etc. As shown in Figure 5(d), KEGG pathways were mainly enriched in the cell cycle and DNA replication. We speculated that the pathways related to the prognostic model might be related to immune infiltration and survival.

### 3.5. Establishment of the Nomogram Model and Decision Tree Model

**Figure 6:**
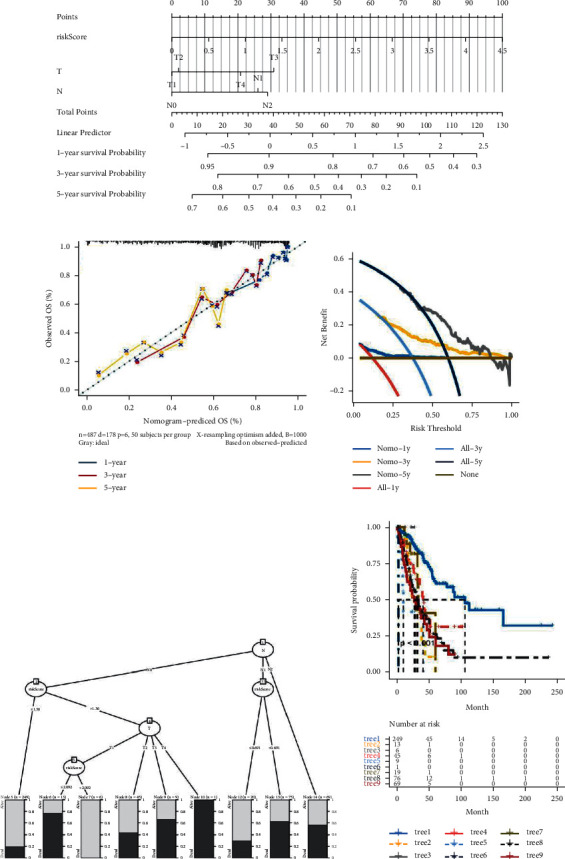
Establishment of the nomogram model and decision tree model. (a) Comprehensive nomogram of 1-, 3-, and 5-year survival rates for patients with LUAD in the training dataset. (b) Calibration curve analysis of the model. (c) DCA curve. (d) The establishment of the decision tree model. (e) Survival analysis of patients in different groups according to the decision tree.

A nomogram model was established using clinical characteristics (T and N) and risk score. Personalized prediction of 1-, 3-, and 5-year survival probability was performed using a comprehensive nomogram (Figure 6(a)). Calibration curve analysis of the model indicated a high degree of consistency between the predicted results and the actual results (Figure 6(b)). The DCA curve indicated that the model had good clinical practicability (Figure 6(c)). Through decision tree analysis of clinical features and risk score, the decision tree was mainly composed of risk score, T, and N (Figure 6(d)). In addition, survival analysis was conducted for patients in different groups according to the decision tree. As shown in Figure 6(e), the survival probability of Tree1 decreased with the increase in time compared with the other eight trees, but the survival probability of Tree1 was relatively high.

## 4. Discussion

The results of this bioinformatics study demonstrated that the survival models (tobacco, T, N, M, stage, sex, age, status, and risk score) were abnormally correlated with T cells and macrophages. The more the risk score was associated with smoking and the higher the stage was, the more severe the LUAD and the more maladjusted the immune system were. Immune infiltration and abnormal expression of immune checkpoint genes in the prognostic model of LUAD were associated with the risk score. In addition, pathways related to the prognostic model may be related to immune infiltration and survival.

LUAD is one of the most common and fatal cancers worldwide. LUAD has significant heterogeneity, and abnormal DNA methylation profiles contribute to tumor heterogeneity and altered immune responses [21]. DNA methylation biomarkers may provide a molecular-level predictor of cancer recurrence risk. In addition, candidate epigenetic biomarkers may provide a theoretical basis for patient stratification and precision medicine, thereby maximizing the chance of successful treatment while minimizing adverse effects [22]. Studies have shown that changes in DNA methylation are frequently observed in LUAD and may play an important role in carcinogenesis, diagnosis, and prediction [23]. In this study, we classified LUAD using the NMF clustering method and obtained 20 DNA methylation regulatory genes to determine the pattern of DNA methylation regulation. In addition, we found that the LUAD survival model (tobacco, T, N, M, stage, sex, age, status, and risk score) was associated with genes involved in regulating DNA methylation.

DNA methylation plays a key role in the “depletion” of cytotoxic T cells associated with tumor progression [24]. The presence of immune cells in the tumor microenvironment is associated with the response of various cancers to immunotherapy [25]. Macrophage polarization is a key regulatory process in tumor progression [26]. Studies have shown that immunogenic chemotherapy enhances the recruitment of CAR T cells to lung tumors and, combined with checkpoint blockade, can improve antitumor efficacy [27]. Moreover, HDAC inhibitors have been reported to enhance T-cell chemokine expression in LUAD and enhance the tumor response to PD-1 immunotherapy [28]. Early clinical tumors lacking memory B cells or with an increased proportion of M0 macrophages are associated with poor prognosis in LUAD [29]. In addition, tumors activated by lipid metabolism tend to have greater immune cell infiltration and a better response to checkpoint immunotherapy [30]. In a previous study on non-small cell lung cancer, invasive immune cells were significantly enriched in tumor tissues of patients, and there was a strong correlation between CD38 and PD-1 expression on CD8+ T cells in tumors [31]. In our study, we found that the LUAD survival model (tobacco, T, N, M, stage, sex, age, status, and risk score) was abnormally correlated with T cells and macrophages. Studies have shown that smoking-related LUAD is associated with other environmental exposures and the field effect in normal adjacent tissues of LUAD [32]. In addition, LUAD patients with STK11 mutant have less immune cell infiltration and a worse prognosis after immunotherapy [33]. Our study showed that the more the risk score was associated with smoking and the higher the stage was, the more severe the disease and the greater the maladjustment of the immune system were in LUAD.

In conclusion, cluster analysis of DNA methylation regulators was helpful to explore the effect of LUAD and immunotherapy. Patients with LUAD with a high risk score showed immune cell inactivation and abnormal immune checkpoint expression. Moreover, the characterization of DNA methylation regulatory patterns served to improve our understanding of the LUAD immune microenvironment and guide the development of a more personalized immunotherapy strategy in the future.

## Figures and Tables

**Table 1:** Clinical features.

Features	*n* = 500	lncRNAs	
		High (*n* = 179)	Low (n = 321)
*Gender*			
Female	270	86	184
Male	230	93	137

*Age*			
<65	219	88	141
≥65	271	91	180
NA	10	3	7

*Status*			
0	318	86	232
1	182	93	89

*Risk score*			
<1	268	0	268
≥1	232	53	179

*Stage*			
I	268	72	196
II	120	60	60
III	79	36	43
IV	25	9	16
NA	8	2	6

*T*			
T1	168	42	126
T2	267	102	165
T3	45	27	18
T4	17	7	10
NA	3	1	2

*N*			
N0	323	106	217
N1	95	41	54
N2	69	30	39
NA	13	2	11

*M*			
M0	332	121	211
M1	24	9	15
NA	144	49	95

*Tobacco*			
No	363	113	250
Yes	119	58	61
NA	18	8	10

*Cluster*			
ClusterA	246	35	211
ClusterB	254	144	110

## Data Availability

All data supporting the findings of this study are reasonably available from the corresponding author upon request.
